# Mark My Words: High Frequency Marker Words Impact Early Stages of Language Learning

**DOI:** 10.1037/xlm0000683

**Published:** 2019-01-17

**Authors:** Rebecca L. A. Frost, Padraic Monaghan, Morten H. Christiansen

**Affiliations:** 1 Language Development Department, Max Planck Institute for Psycholinguistics, Nijmegen, the Netherlands; 2 Psychology Department, Lancaster University; Department of English Language and Culture, University of Amsterdam; and Max Planck Institute for Psycholinguistics, Nijmegen, the Netherlands; 3 Department of Psychology, Cornell University; Interacting Minds Centre and School of Communication and Culture, Aarhus University; and Haskins Laboratories, New Haven, Connecticut

**Keywords:** language learning, speech segmentation, grammatical categorization, statistical learning

## Abstract

High frequency words have been suggested to benefit both speech segmentation and grammatical categorization of the words around them. Despite utilizing similar information, these tasks are usually investigated separately in studies examining learning. We determined whether including high frequency words in continuous speech could support categorization when words are being segmented for the first time. We familiarized learners with continuous artificial speech comprising repetitions of *target words*, which were preceded by high-frequency *marker words.* Crucially, marker words distinguished targets into 2 distributionally defined categories. We measured learning with segmentation and categorization tests and compared performance against a control group that heard the artificial speech without these marker words (i.e., just the targets, with no cues for categorization). Participants segmented the target words from speech in both conditions, but critically when the marker words were present, they influenced acquisition of word-referent mappings in a subsequent transfer task, with participants demonstrating better early learning for mappings that were consistent (rather than inconsistent) with the distributional categories. We propose that high-frequency words may assist early grammatical categorization, while speech segmentation is still being learned.

There are many tasks that learners must master in order to achieve linguistic proficiency, including discovering individual words from speech, and recognizing that these words belong to different grammatical categories. Yet, speech contains no absolute acoustic cues to word boundaries (e.g., Aslin, Woodward, LaMendola, & Bever, 1996), nor are there perfectly reliable indicators of grammatical category membership (e.g., Monaghan, Christiansen, & Chater, 2007). Learners therefore must look to additional sources of information to succeed at these tasks. One source that learners can draw upon is the distributional information contained in speech: Statistical patterns of co-occurrence (e.g., between phonemes, syllables, words) are ubiquitous in language, and can shed critical light on its structure both in terms of what constitutes words (Bortfeld, Morgan, Golinkoff, & Rathbun, 2005) and what constitutes grammatical categories (Monaghan et al., 2007). Here, we examine the possibility that the same distributional cue (high frequency marker words) can assist multiple tasks in language learning at the same time; statistical speech segmentation, and categorization of the segmented items.

Past research has highlighted learners’ remarkable aptitude for computing transitional probabilities between syllables in speech, and using them to help infer word boundaries in both artificial (e.g., Aslin, Saffran, & Newport, 1998; Saffran, Aslin, & Newport, 1996; Saffran, Newport, Aslin, Tunick, & Barrueco, 1997) and natural languages (Pelucchi, Hay, & Saffran, 2009). Further, studies have shown that learners can draw on transitional information for speech segmentation from infancy onward, perhaps even before they know the meaning of a single word in the language (e.g., Saffran et al., 1996; Thiessen & Saffran, 2003) with sensitivity to distributional structure present possibly even from birth (Teinonen, Fellman, Näätänen, Alku, & Huotilainen, 2009).

Since distributional regularities exist at multiple levels of language abstraction, it follows that statistical regularities in speech may benefit a range of language learning tasks in addition to speech segmentation. Indeed, learners have been shown to be capable of using the statistical information contained in speech to acquire rule-like linguistic regularities with varying levels of complexity (e.g., Endress & Bonatti, 2007; Endress & Mehler, 2009; Gerken, 2010; Gómez, 2002; Lany & Gómez, 2008; Lany, Gómez, & Gerken, 2007; Marcus, Vijayan, Bandi Rao, & Vishton, 1999; Mueller, Bahlmann, & Friederici, 2008, 2010; Peña, Bonatti, Nespor, & Mehler, 2002). Further, recent research has suggested that learners can learn structural generalizations even while they are still learning to segment speech (Frost & Monaghan, 2016).

Given learners’ demonstrable sensitivity to the distribution of linguistic patterns, it follows that items appearing in speech with a higher frequency than others might have a particularly important role in language learning—especially since high-frequency items are more easily perceived than lower-frequency words of similar length (Morgan, Shi, & Allopenna, 1996; Zipf, 1935), and provide more reliable co-occurrence information than their less frequent counterparts (Monaghan, Chater, & Christiansen, 2005). Indeed, recent research has suggested that the presence of high-frequency words in speech may be advantageous for language acquisition, particularly for speech segmentation (Altvater-Mackensen & Mani, 2013; Bortfeld et al., 2005; Kurumada, Meylan, & Frank, 2013; Mersad & Nazzi, 2012).

One way that frequently occurring words could assist speech segmentation is by providing learners with helpful information about the boundaries of words that surround them, such that these high-frequency words operate as *anchors*, around which further language acquisition can occur (Bortfeld et al., 2005). This “anchor effect” has been suggested to facilitate interplay between top-down lexical segmentation, drawing on learners’ knowledge of known words, and bottom-up identification of the edges of unfamiliar items, drawing on the statistics of the input (e.g., Conway, Bauernschmidt, Huang, & Pisoni, 2010), thereby helping learners to identify words in speech. We can consider how this might work by taking the sentence *youeatthebiscuityetyoudrinkthetea*: Upon hearing this sentence, a learner could recognize high-frequency words *you* and *the,* and use these to discern information about the words that surround them. In this instance, recognizing the word *you* could help the learner to identify the way in which the succeeding (*eat*, *drink*) and preceding (*yet*) words begin and end, respectively, thus facilitating segmentation. Critically though, some of the speech will remain unsegmented (*biscuityet*), meaning that high-frequency words do not entirely solve the task of speech segmentation—learners must extract the remaining words through further processing, such as computing the transitional probabilities of syllables within words, and inferring word boundaries at points where the subsequent syllable is difficult to predict (e.g., Saffran et al., 1996), or exploiting the broad array of prosodic cues that support segmentation (Curtin, Mintz, & Christiansen, 2005; Frost, Monaghan, & Tatsumi, 2017; Mattys, White, & Melhorn, 2005; Monaghan, White, & Merkx, 2013; Turk & Shattuck-Hufnagel, 2000).

In a seminal study, Bortfeld, Morgan, Golinkoff, and Rathbun (2005) demonstrated that 6-month-old infants were better able to identify new words in short utterances of speech when they appeared alongside high frequency words (e.g., their own name, and the word “mommy”) compared with when they appeared next to an unfamiliar item, providing early evidence that learners may draw on highly familiar high-frequency words to help them during speech segmentation. Further evidence for the anchor word effect has since been found for both infant (Mersad & Nazzi, 2012) and adult learners (Cunillera, Càmara, Laine, & Rodríguez-Fornells, 2010). Crucial support comes from Cunillera, Laine, and Rodríguez-Fornells (2016), who documented the neural signature of this effect in adults, with results indicating that anchor words elicited greater stimulus-preceding negativity (a marker of expectation for subsequent input) in the learners’ electroencephalography (EEG) data, in keeping with the notion that learners harbored an expectation for a particular word following a highly familiar high frequency word.

Additional support for the anchor word effect can be found in literature on computational modeling of speech segmentation. Monaghan and Christiansen (2010) examined the possibility that highly frequent words may assist with natural language acquisition using their PUDDLE model of speech segmentation, which they applied to natural language corpora of child-directed speech taken from the CHILDES database (MacWhinney, 2000). The model began by treating each utterance as a potential word, and then segmenting other utterances when they contained previously stored word candidates. In the model, high-frequency words were extracted quickly and, critically, these words were used to help segment the rest of the speech input. Together with the behavioral data, these findings provide converging evidence that high-frequency words may assist language acquisition by facilitating segmentation, particularly when they border entirely unfamiliar words (Bortfeld et al., 2005).

Of equal importance is the possibility that, in addition to helping with speech segmentation, high-frequency words may also help inform the formation of grammatical categories (Valian & Coulson, 1988). In the example sentence “*youeatthebiscuityetyoudrinkthetea,” you* reliably precedes verbs (*eat*, *drink*), while *the* reliably precedes nouns (*biscuit*, *tea*), in keeping with Mintz’s (2003) observation that pronouns often precede verbs but seldom precede nouns, while determiners often precede nouns but only rarely verbs. Crucially, Mintz (2003) suggested that learners could exploit these co-occurrences for grammatical categorization (see also St. Clair, Monaghan, & Christiansen, 2010). Thus, it is possible that high frequency words can provide learners with valuable grammatical information about the words that follow them in speech, as well as helping with their initial discovery.

Interestingly, Monaghan and Christiansen (2010) noted a substantial overlap between the high frequency words that were first extracted by their PUDDLE model of speech segmentation and words that were found to be useful for identifying grammatical categories in previous studies of child-directed speech (Monaghan et al., 2007), indicating that some of the same high frequency words may assist processing for different tasks during natural language acquisition, perhaps at the same time. To test the extent to which speech segmentation could support word learning, Graf Estes, Evans, Alibali, and Saffran (2007) and Hay, Pelucchi, Graf Estes, and Saffran (2011) exposed English-learning infants to continuous speech in an unfamiliar language, then tested their ability to use words from that speech to label objects on a word–picture mapping task. They demonstrated that speech segmentation provided learners with a valuable opportunity to learn about word forms, which can immediately be linked to meaning. But what kind of information do learners acquire at the point of segmentation about the identity of potential word candidates and their possible *grammatical roles* in speech?

Learners’ capacity to exploit the link between distributional and grammatical categories has been subject to extensive theoretical debate. For instance, Chomsky (1965) proposed that grammatical categories are innately specified, while Pinker (1987) suggested that semantic features are innately specified, with grammatical categories being mapped onto the relevant semantic features that are realized within the grammar of the particular language.^1^ Alternatively, it may be that grammatical categories can be entirely derived from the complex distributional statistics of their usage (Bates & MacWhinney, 1982; Redington, Chater, & Finch, 1998). Yet, the way that distributional categories (such as words succeeding *the* vs. words succeeding *you*) relate to syntactic grammatical distinctions (such as nouns and verbs) has rarely been explicitly investigated under the controlled conditions of artificial language learning studies. It may be that the semantic distinctions must be present while language learners are initially exposed to distributional categories, as in studies by Frigo and MacDonald (1998) and Braine et al. (1990). Alternatively, it may be that abstract distributional categories can be acquired (e.g., Gerken, Wilson, & Lewis, 2005; Kiss, 1973; Mintz, 2002; Reeder, Newport, & Aslin, 2013) then later mapped onto distinct syntactic categories.

Reeder, Newport, and Aslin (2013) provided a comprehensive analysis of the way that learners form abstract distributional categories from linguistic input; in a series of studies, they trained adults on an artificial grammar and varied the presence of distributional information on a variety of dimensions, including the number of linguistic contexts a word could appear in, the density of these contexts in the language input, and the overlap between words and contexts. Their findings demonstrated that learners can draw on distributional information alone to inform categorization of lexical items, and can even use it to generalize across gaps in their input.

More recently, Lany (2014) tested the way in which learners map distributional information onto semantic categories (animals and vehicles). In this study, 22-month-old infants heard a familiarization stream comprising novel determiners and novel nouns, which were presented alongside one another in segmented speech. Determiners and nouns were split into two categories, which were paired together such that particular determiners preceded particular nouns (i.e., aX, and bY), and items in paired categories appeared together probabilistically within the speech stream (i.e., with 75% co-occurrence, to reflect noise in natural language), cueing distributional category membership. Following familiarization, infants completed a word–picture mapping task, during which nouns were paired with images of unfamiliar animals and vehicles. Mappings were probabilistic, with X and Y nouns predominantly labeling animals and vehicles, respectively. The data indicated that the co-occurrence statistics in the familiarization stream may have informed the subsequent formation of semantic categories; helping learners to learn the labels of new animals and vehicles (though this was only the case for infants with higher scores on an independent measure of grammar development). Critically, since Hay et al. (2011) demonstrated that *speech segmentation* provides learners with an opportunity to learn word candidates, it is possible that learners would demonstrate evidence of mapping distributional structure to grammatical categories even when exposed to continuous speech.

Bringing together the literature on high frequency words, statistical speech segmentation, and grammatical categorization, the present study examined whether the same high-frequency marker words could influence statistical speech segmentation and grammatical categorization simultaneously. We trained adults on a continuous artificial speech stream consisting of *target words* and *marker words*, which distinguished target words into two distributional categories through co-occurrence, with certain marker words always preceding words in certain categories. We tested whether the high-frequency marker words helped learners to identify the target words that surrounded them in speech (speech segmentation). We also examined whether learners used these same high-frequency words to discern (explicitly) that target words belonged to different abstract categories (categorization) according to distributional co-occurrence information in the speech stream (Mintz, 2003; Monaghan et al., 2007).

In a subsequent test, we assessed the extent to which learners’ distributional category knowledge implicitly influenced grammatical category learning, by applying the language to a cross-situational learning task, which mapped target words onto actions or objects (see Graf Estes et al. (2007) for evidence that learners can map new words onto recently segmented items). Cross-situational word learning tasks require learners to form word/referent associations over repeated exposure to scenes that over time provide useful statistical cues, but are individually ambiguous. Such tasks take into account the dynamic nature of the language learning environment: each spoken word could have a virtually infinite number of possible referents (Quine, 1960), but learners are likely to encounter repeat co-occurrences of particular words and referents over multiple learning instances (Gleitman, 1990; Horst, Scott, & Pollard, 2010; Pinker, 1989; Smith, Smith, & Blythe, 2011). Learners can exploit these *cross-situational statistics* in order to learn word-referent mappings (Fitneva & Christiansen, 2011, 2017; Monaghan & Mattock, 2012; Monaghan, Mattock, Davies, & Smith, 2015; Smith & Yu, 2008; Yu & Smith, 2007), and can use them to acquire both item and category labels for objects (Chen, Gershkoff-Stowe, Wu, Cheung, & Yu, 2017). In an extension of these prior cross-situational learning tasks, we required participants to draw on associations made between words and action/object referents over multiple trials. Critically, we required learners to pair words with referents in a way that was either consistent with the categories defined in the continuous speech (with words from each distributional category being used as either nouns or verbs, labeling objects and actions, respectively) or inconsistent (with half of the words from each distributional category being used as nouns and the other half as verbs).

In a related pilot study, Frost, Monaghan, and Christiansen (2016) trained participants on a continuous speech stream in which target words were presented alongside (category-denoting) markers, and tested transfer of distributional category learning to a word–picture mapping task, which required participants to map words from these distributionally defined categories to pictures of actions and objects, with mappings being either consistent or inconsistent with the distributional category distinction. The pilot study found some evidence to suggest word-object/action mapping was influenced by distributional categorization, but this was weak (evidenced by a marginally significant effect). Further, this effect was only observed in the first testing block, and diminished over the course of task—suggesting that, if distributional information is used to prepare the learner for potential category distinctions in their vocabulary, then this may only exert an effect during the initial stages of learning. We therefore examined whether transfer effects are observed early in the cross-situational learning task, as well as throughout it. The current study design improved on this earlier study by increasing the salience of the distinction between object and action referents in the transfer task by using animated movements rather than pictures of actions. We anticipated that the current study would show a larger effect of marker word categorization than was seen in the pilot, and we expected that this effect may dissipate over time—as associative information about words and referents increases.

We hypothesized that high-frequency words operating as markers to word boundaries might also assist with speech segmentation (Altvater-Mackensen & Mani, 2013; Bortfeld et al., 2005; Kurumada et al., 2013; Mersad & Nazzi, 2012). Additionally, we hypothesized that these marker words might also simultaneously constrain learning about the role of other words in the language by contributing to early formation of grammatical categories (Lany, 2014). If participants do form grammatical categories based on the distribution, then we expect knowledge of these categories to interfere with their application of the language during the transfer task, when the latter is inconsistent with learned category information. Specifically, we expect that this knowledge will impede participants’ ability to put target words from the same grammatical category to different uses (i.e., using them to label both nouns and verbs, rather than just nouns, or just verbs). We anticipated that this effect would be observed most clearly during the early stages of training on the transfer task. Alternatively, if distributional information can only be used in concordance with semantic information to generate information about the syntactic categories of referents (e.g., Pinker, 1987), then prior learning of distributional categories will not be seen to affect acquisition of word-referent mappings in the transfer task. A further possible cause for a lack of effect of distributional categories on syntactic categorization could be that speech segmentation does not initially permit categorization information to permeate through the learner’s language structure.

## Method

### Participants

Participants were 48 adults (16 males, 32 females), all students at Lancaster University, with a mean age of 19.10 years (range = 18–22 years). All participants were native-English-speakers, with no known history of auditory, speech, or language disorder. Participants were paid £3.50 or received course credit.

### Design

The experiment used a between-subjects design, with two conditions of training type: *markers and no markers*. These conditions varied the number of marker words present in the speech and either contained no marker words, or one marker word per category. Participants were randomly allocated to one of these conditions, with 24 participants receiving each type of training. All participants completed the same battery of tests after training; knowledge of the experimental language was tested immediately with tasks assessing speech segmentation and distributional categorization. A transfer task then put the familiarized language to use, to see whether participants’ distributional category knowledge for the target words shaped the way they used those targets as labels for actions and objects. For this task, participants were further subdivided into two groups for whom objects and actions were labeled in a way that was either *consistent* or *inconsistent* with the distributional categories (each *N* = 12). This subdivision was crossed with the markers/no markers conditions, such that half of the participants in each group received consistent labels, and the other half received inconsistent labels. Note that the no markers condition does not relate meaningfully to the consistent versus inconsistent distinction, but was included as an additional control to ensure that any effects observed for the markers condition were not due to biases in participants’ responses to individual items.

Sample size was designed with respect to our key experimental test of transfer from a speech segmentation task to a label-mapping task. We assessed the results from Graf Estes et al. (2007) and Frost et al. (2016)—studies in the literature which most closely resembled the current study design. Graf Estes et al. (2007) demonstrated a transfer effect of η_p_^2^ = .16 in a two-way mixed ANOVA for an infant looking study to stimuli that either matched or mismatched object-labels, with 14 participants in each condition (conditions were *word-transfer* and *nonword-transfer*). Post hoc power was .88, assuming zero correlation between matching and mismatching conditions (an intercorrelation would increase power further). Graf Estes et al. (2007) employed one within-subject and one between-subjects factor, whereas our design required two between-subjects factors. For a similar effect size, a priori power = .84 with 24 participants distributed across the marker word and no marker word conditions (with *N* = 12 in the consistent and inconsistent mapping conditions). For the pilot study by Frost et al. (2016), three different between subject marker word conditions each had 24 participants, subdivided into groups receiving consistent and inconsistent mappings (*N* = 12 for each subgroup). The size of the effect of marker word consistency on transfer to the early stages of word learning was η_p_^2^ = .124, with power = .81.

In the current study we analyzed results using linear mixed effects models which can enhance power further (Brysbaert & Stevens, 2018). As there were no similar studies using linear mixed effects analysis, we were unable to determine the required sample size for the study a priori. We thus report post hoc power for each of the measures we assessed below, generated using the simR package (Green & MacLeod, 2016), as recommended by Brysbaert and Stevens (2018).

This study received ethical approval from the Faculty of Science and Technology Research Ethics Committee at Lancaster University.

The experimental language and the stimuli and procedure for each of the tasks are outlined below.

### Materials

#### Stimuli

Speech stimuli were created using the Festival speech synthesizer (Black, Taylor, & Caley, 1990). The language was created from a set of 20 syllables (*no, ro, fo, to, li, gi, ni, ka, ma, sa, za, fe, te, re, de, ve, mu, zu, pu, bu*), which were drawn upon and combined pseudorandomly to create eight bisyllabic *target words* (e.g., *samu, noli, nide, fezu, tero, buza, kato, mave),* and two monosyllabic *marker words (*e.g., *fo, pu*), which preceded target words in the speech stream. Phonemes used for targets and marker words contained both plosive and continuant sounds. There was no repetition of consonants or vowels within target words. Each target word lasted approximately 500 ms, and each marker word lasted approximately 250 ms. Eight transitions between words were omitted from the no markers familiarization stream (e.g., *noli* never succeeded *samu*), in order to create a set of nonwords involving syllable transitions not seen by participants in either training condition (see Segmentation Test section for details).

The eight target words were arbitrarily split into two equal categories (*A* and *B),* with four words in each. Category membership was denoted only by the co-occurrence of target words and marker words in the continuous speech stream: in the markers condition, one marker word reliably preceded words from each category (e.g., *fo* preceded A words, whereas *pu* preceded B words). The speech stream for the no markers condition contained target words only, meaning participants in this condition received no information regarding the category membership of the target words, therefore we would not expect them to demonstrate such knowledge at test.

Six versions of the language were generated by randomly assigning syllables to positions within words and marker words, to control for possible preferences for certain syllables in certain positions, and preferences for particular dependencies between syllables not due to the statistical structure of the sequences (Onnis, Monaghan, Richmond, & Chater, 2005). These versions were counterbalanced across each of the two training conditions.

#### Training

A continuous stream of synthetic speech was created using the Festival speech synthesizer (Black et al., 1990) by concatenating target words and marker words (see Table 1). For the no markers control condition, the speech stream comprised target words only, and lasted approximately 280 seconds. For the markers condition, the speech stream comprised target words plus marker words, and lasted approximately 420 s. In both conditions, the eight target words were each presented 150 times with no immediate repetition, and speech was continuous, with no pauses between words. Speech streams had a 5-s fade in and out so that the onset and offset of speech could not be used as a cue to the word boundaries or language structure.[Table-anchor tbl1]

#### Segmentation test

To test segmentation, we created a two-alternative forced-choice task, which examined participants’ preferences for words versus nonwords. Nonwords were bisyllabic items that comprised the last syllable of one target word and the first syllable of another (e.g., *muno* formed from *samu* and *noli*). We used nonwords (items which did not occur during familiarization) in order to make comparisons across the different conditions. For the no markers condition, particular transitions between target words were withheld from the speech stream, and nonwords were formed from the resulting syllable combinations of the omitted transitions (so for this group nonwords are comparable with part-words in classic instances of this paradigm). The same nonwords were used at test for the markers condition, and these did not occur in the familiarization speech for an additional reason: A marker word intervened between pairs of target words. Note that it would not have been possible to use part-words which spanned word boundaries as in Saffran, Aslin, and Newport’s (1996) studies of speech segmentation, because part-words did not occur in a comparable way across conditions: Part-words in the markers condition would comprise a fragment of a target word and a marker word, in the no markers condition, they would have to comprise fragments of two target words. In our task, preference for selecting words over nonwords would indicate that participants had successfully distinguished target words from competitor nonword syllable sequences.

Eight test pairs were constructed by matching each target word with a corresponding part-word (e.g., *samu* vs. *muno*), and items in each test pair were separated by a 1-s pause. Test pairs were each presented twice, giving 16 test items in total. Items were presented in random order, and correct responses occurred an equal number of times in the first and second position within pairs.

#### Categorization test

To test abstract categorization (i.e., categorization based on the distributional information about the co-occurrence of markers and targets), we created an explicit similarity-judgment task that contained pairs of target words. Twelve test pairs contained items from the same category (as determined by the marker words that preceded them in speech), with six test pairs containing two Category A words, and six test pairs containing two Category B words. There were also 12 mixed test pairs, which contained one word from each category (so, one A word and one B word), giving 24 test pairs in total.

#### Transfer of category knowledge test

To test whether knowledge of distributional categories constrained participants’ learning of word-referent mappings, we created a cross-situational word-picture/action mapping task, which provided a grammatical category distinction (i.e., nouns and verbs) onto which the target words could map. On each trial, participants heard a sentence comprising two targets, then saw two visual scenes, with each scene containing a shape undertaking an action (with no duplication of shapes and actions on individual trials). Participants stated which of the two visual scenes the sentence described (see Monaghan et al., 2015 for a similar experimental design of cross-situational learning but without the preceding segmentation task, and see the Procedure section for more information about this task). There were four images of shapes, each printed in black on a grey background, taken from Fiser and Aslin (2002). There were four actions that these shapes could perform: rotate, bounce, swing, and shake, selected from the series of actions used by Monaghan et al. (2015).

Of the eight target words, four were paired with different shapes, and four were paired with different actions. Sentences were constructed to describe possible noun–verb combinations such that each sentence contained two target words, with one word referring to the shape, and one word referring to the action it was undertaking.

**Figure 1 fig1:**
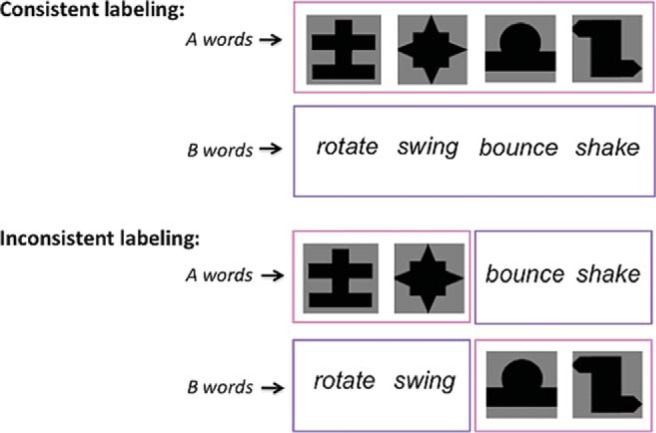
Consistent versus inconsistent word-action/object mappings.

Critically, for half of participants, word-action/shape pairings were *consistent* with the distributionally defined categories heard during training, such that all A words appeared with shapes and all B words appeared with actions. For the remaining participants, pairings were *inconsistent*: two A words and two B words were paired with shapes, and two A words and two B words were paired with actions (see Figure 1).[Fig-anchor fig1]

There were six versions of the language, presentation of which was counterbalanced across participants. Each version of the language used a different set of images for this task, which were selected at random from a set of eight novel shapes (taken from Fiser & Aslin, 2002). Particular objects and actions co-occurred an equal number of times, to prevent formation of associations between particular objects and actions, and to minimize unintentional co-occurrences between nouns and actions and between verbs and objects. Word-referent pairings differed across the six different versions of the language, to control for potential preferences for linking certain sounds to particular objects or actions.

#### Vocabulary test

Finally, we created a vocabulary test to assess exactly which word-object/action mappings participants had learnt. This task contained 16 two-alternative forced-choice trials, with two trials for each target word. Trials assessing learning of nouns (word-object mappings) contained static images of two objects: the target object and one other trained object that acted as a foil. For trials assessing learning of verbs (word-action mappings), we introduced a new shape to prevent participants’ responses from being influenced by knowledge of shapes. On verb trials, participants saw two scenes containing a new shape (presented alongside one another onscreen, as in the noun trials) with the shape performing a different action in each scene. On each trial, after 5 s (while the scenes were still onscreen) a target word was presented auditorily, and participants selected which of the two objects, or actions, the target was referring to. Pairing of shapes and actions was pseudorandomized such that each shape and action featured an equal number of times over the course of the task: twice as the correct referent (one to the left, and once to the right), and twice as the alternative (once to the left, and once to the right).

### Procedure

Before hearing the familiarization speech, participants were instructed to pay attention to the language and think about the possible words it may contain. Participants were tested immediately after training. Testing was structured such that all participants received the tasks in the same order: Participants completed the segmentation test first, followed by the categorization test, then the cross-situational transfer test. Tasks were programmed using EPrime 2.0, with instructions appearing onscreen before each task began.

For the segmentation test, participants were instructed to listen to each test pair (a word and a nonword) then select which item best matched the language they had just heard, responding “1” for the first or “2” for the second sequence on a computer keyboard.

For the categorization test, participants were instructed to listen to each test pair (two words from the same/different categories), then rate how similar they thought the role of the items was in the familiarization stream. Participants were required to respond on a computer keyboard using a 6-point Likert-scale, with 1 = *extremely different roles* and 6 = *very similar roles*. If participants have formed categories based on the co-occurrence of target words and markers, then pairs of items taken from the same category should receive higher similarity ratings than mixed pairs (see Frost et al., 2016, for a variant of this paradigm that demonstrates abstract category learning is possible).

**Figure 2 fig2:**
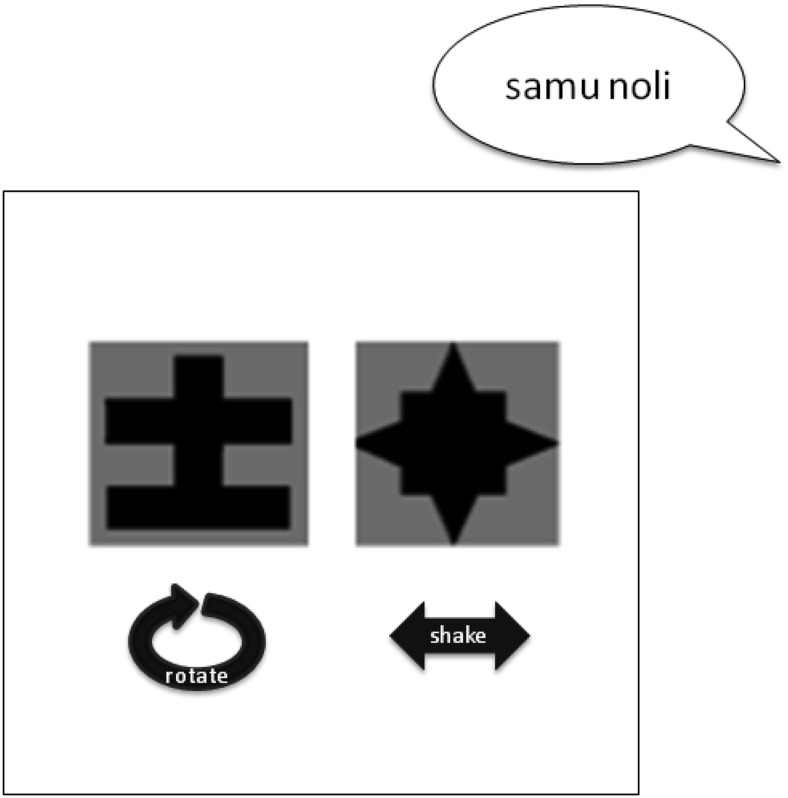
An example trial on the cross-situational learning task, with two shapes performing unique actions, presented alongside a sentence that describes one of these pairs.

For the cross-situational task, on each trial participants saw two scenes containing different objects performing different actions. After 5 s (while the scenes were still onscreen) participants heard a sentence comprising two target words. The sentence described one of the two scenes, with one word referring to the action, and one to the object. When the sentence had finished, participants were instructed to indicate via key press whether the sentence described the scene on the left or the right of the screen, by pressing “1” for the left and “2” for the right. The next trial began after participants had provided their response. An example trial is shown in Figure 2.[Fig-anchor fig2]

The probability of co-occurrence between a noun (noun_*i*_) and its target object (object_i_) was *p*(object_i_|noun_i_) = 1, whereas co-occurrence between a noun and another distractor object was *p*(object_j_|noun_i_) = 0.33, and co-occurrence probability between a noun and each action was *p*(noun_i_|action_j_) = 0.25. Similarly, verb to target action co-occurrence probabilities were *p*(action_i_|verb_i_) = 1, verb to other action probabilities in the distractor scene were *p*(action_j_|verb_i_) = 0.33, and verb and object co-occurrence probabilities were *p*(object_i_|verb_i_) = 0.25. Over the course of the task, we expected that learners would draw on these co-occurrence statistics in order to learn word-referent mappings.

There were six blocks, each containing eight learning trials. Within each block, each image and motion occurred four times—twice in the target scene and twice in the alternative (foil) scene. Each word occurred twice in each block. The left/right position of the referent and alternative scene was pseudorandomized such that each scene appeared once in each position.

To avoid providing additional cues for the role of words in the language, the presentation order for nouns and verbs was counterbalanced such that half of the sentences in a block followed a noun–verb order, and the other half followed a verb–noun order. Thus, this task used free word-order, such that grammatical categories of words were defined only by their prior co-occurrence with the marker words, and not in terms of the sentence position.

If prior category knowledge was influencing performance on this task, then participants should find it easier to use words from each of the categories consistently (i.e., all A words labeling objects) than inconsistently (i.e., some A words labeling objects, but some A words labeling actions). Thus, the key interaction of interest involves condition and consistency, which would reflect transfer of distributional category knowledge.

For the vocabulary test, on each trial participants saw the two scenes then heard a target word and selected via key-press whether the word referred to the scene on the left or the right of the screen (pressing “1” for left and “2” for right, as in the cross situational task). The next trial began after participants had responded. There were 16 trials in total, with two trials for each target word. The order of noun and verb trials was randomized, and the left/right position of the referent and foil alternative was balanced in the testing block.

Training and testing stimuli were presented at a comfortable volume, through closed-cup headphones. All participants were tested individually in an isolated booth, and the entire session lasted for approximately 30 min.

## Results

We first report the results of the segmentation task, investigating the effect of marker words on participants’ ability to individuate words from the speech, relative to the no markers control group. We then present the results for the categorization test, which assesses whether participants encoded category information about the words on the basis of their co-occurrence with markers. Finally, we report the key analysis for the study, which is whether category information defined by the marker words can have an implicit effect on participants’ ability to use words as nouns and verbs in the word-object/action transfer task. For this test, we first report transfer effects seen during early stages of learning (consistent with learning effects observed in Frost et al., 2016), we then report the results across the whole task, followed by the measures of learning of nouns and verbs.

### Segmentation

One-sample *t* tests were performed on the segmentation data (proportion correct responses) to compare performance with chance. Performance was significantly above chance for both no markers (*M* = .740, *SE* = .042), *t*(23) = 5.658, *p* < .001; and markers (*M* = .666, *SE* = .028), *t*(23) = 5.914, *p* < .001, indicating that participants in both conditions were able to identify individual words from the speech stream.

Generalized linear mixed effects analysis was performed on the data (Baayen, Davidson, & Bates, 2008), modeling the probability (log odds) of response accuracy on the segmentation test considering variation across participants and materials. The model was built incrementally, and was initially fitted with the maximal random effects structure that was justified by the design, with random effects of subjects, particular test-pairs, and language version (to control for variation across the randomized assignments of phonemes to syllables). Random slopes were omitted if the model failed to converge with their inclusion (Barr, Levy, Scheepers, & Tily, 2013). We then added condition (markers, no markers) as a fixed effect, and considered its effect on model fit with likelihood ratio test comparisons. There was no significant effect of condition (model fit improvement over the model containing random effects: χ^2^(1) = 2.850, *p* = .091, power = .46, 95% CI [.39, .53]), indicating participants in both conditions performed at a statistically similar level (difference estimate = −.459, *SE* = .27, *z* = −1.70, see Frost et al., 2016 for a similar observation in a pilot of this task). See Table 2 for a summary of the final model, and see the online supplemental materials for a visualization of the data for this task.[Table-anchor tbl2]

### Categorization

Linear mixed effects analysis was performed on the data for the categorization test (Baayen et al., 2008), to model the probability (log odds) of providing different similarity ratings for test pairs containing items taken from the same versus different distributional categories. The model was built incrementally, and was initially fitted specifying random effects of subjects, test trial, and language version. Random slopes were omitted if the model failed to converge with their inclusion. We then added fixed effects incrementally, and these were retained if they contributed significantly to model fit.

We first added condition (markers, no markers) as a fixed effect, and considered its effect on model fit with likelihood ratio test comparisons (compared with a model containing just random effects). There was a significant effect of condition, χ^2^(1) = 4.540, *p* = .033, power = .50, 95% CI [.43, .57], with the no markers group giving significantly higher similarity ratings overall (*M* = 3.876, *SE* = .116) compared with the markers group (*M* = 3.518, *SE* = .116). There was no significant effect of test-pair type, χ^2^(1) = 0.038, *p* = .846, power = .05, 95% CI [.02, .09], and there was no significant interaction between condition and test-pair type, χ^2^(1) = .051, *p* = .822, power = 0, 95% CI [0, .02], determined through comparing fit for a model containing fixed effects for test pair type and condition with a model containing fixed effects plus the interaction term. These findings indicate that there was no difference in similarity ratings for test-pairs containing the same versus different categories across the conditions. See Table 3 for a summary of the final model, and see the online supplemental materials for a visualization of the data for this task.[Table-anchor tbl3]

### Transfer of Category Knowledge

One-sample *t* tests were performed on the data for the transfer task to compare performance to chance (taken as .5 in accordance with the number of options available per trial at test). Performance was significantly above chance for no markers (*M* = .563, *SE* = .019), *t*(23) = 3.226, *p* = .004, and for markers (*M* = .575, *SE* = .026), *t*(23) = 2.844, *p* = .009, indicating that participants in both conditions could draw on the cross-situational statistics, and use them to learn the word-action/object mappings over the course of the task.

We distinguished overall accuracy from initial performance on the transfer task separately in a two-stage preplanned analysis, focusing initially on the first block of testing (Test 1), to examine the way participants’ immediate responses were influenced by their training (see Frost et al., 2016).

#### Performance at Test 1

Generalized linear mixed effects analysis was performed on the response data from the first block of training, modeling the probability (log odds) of response accuracy considering variation across participants and materials. The model was built incrementally, and was initially fitted specifying random effects of subjects, trial, particular target items (Target 1 and Target 2 in each sentence), word order (noun–verb or verb–noun) and language version, and random slopes were omitted if the model failed to converge with their inclusion. We then sequentially added condition (markers, no markers) and consistency (consistent/inconsistent) as fixed effects, followed by the interaction term, and considered their respective effects on model fit with likelihood ratio test comparisons. Because only the markers group received distributional category cues, and only the inconsistent group encountered conflict between distributional categories and the categories on this task, transfer effects here would be evidenced through an *interaction* between these two variables.

There was no significant effect of condition on overall performance at Test 1 (model fit improvement over the model containing random effects: χ^2^(1) = .833, *p* = .361, power = 0, 95% CI [0, .02], indicating participants in the marker and no marker groups were not significantly different overall (difference estimate = .258, *SE* = .282, *z* = .916). As predicted, there was also no significant main effect of consistency (model fit improvement over the model containing random effects: χ^2^(1) = .060 *p* = .806, power = 0, 95% CI [0, .02], indicating participants receiving consistent and inconsistent labeling performed at a statistically similar level overall (difference estimate = −.070, *SE* = .284, *z* = −.245). This result was expected given that consistent or inconsistent labeling is only functionally relevant for participants in the marker group.

**Figure 3 fig3:**
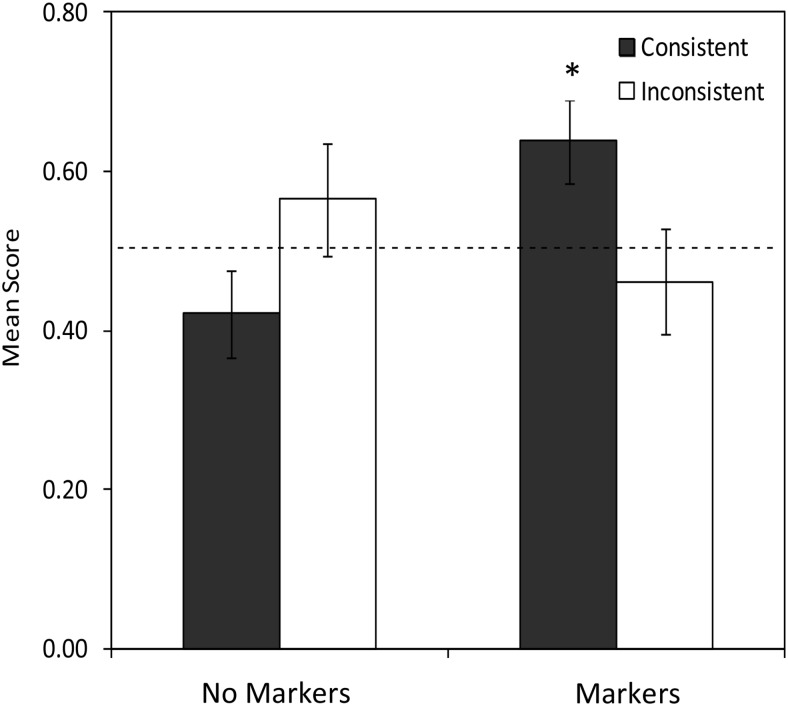
Mean scores on the cross-situational learning task at Test 1 (proportion correct), with standard error. * Asterisks indicate statistical significance.

Critically, the key interaction between condition and consistency was significant (model fit improvement over a model containing main and random effects: χ^2^(1) = 7.114, *p* = .008, difference estimate = −1.438, *SE* = .528, *z* = −2.722, power = .76, 95% CI [.69, .81]). Further analysis indicated that this interaction was driven by better performance for participants receiving consistent (*M* = .638, *SE* = .052) compared with inconsistent (*M* = .461, *SE* = .066) labeling in the markers group, *t*(22) = 2.101, *p* = .047, while participants receiving inconsistent (*M* = .565, *SE* = .071) versus consistent (*M* = .421, *SE* = .054) labeling in the no markers group were not significantly different, *t*(22) = −1.615, *p* = .121. See Table 4 for a summary of the final model, and see Figure 3. [Table-anchor tbl4][Fig-anchor fig3]

To further explore the effects of consistency on performance at Test 1, one-sample *t* tests were conducted for each condition, comparing performance with chance. Only participants in the markers group receiving consistent training performed significantly above chance at Test 1, *t*(11) = 2.623, *p* = .024 (scores for markers inconsistent and no markers consistent and inconsistent were all *p* > .171), further suggesting that learners’ prior distributional categories shaped their application of the language in the transfer task.

#### Overall performance

In subsequent analysis, we examined performance across the task as a whole. Generalized linear mixed effects analysis was performed on the data from the entire task, modeling the probability (log odds) of response accuracy on the transfer test as a whole considering variation across participants and materials. The model was initially fitted specifying random effects of subjects, target items (Target 1 and Target 2—the first and second target in each sentence), word order (noun–verb or verb–noun) and language version (so, random effects were the same as those used for analysis of performance at Test 1, but without trial which would be confounded with test time). We then added condition, consistency, and test time as fixed effects, followed by the interaction terms for these variables, and considered their respective effects on model fit with likelihood ratio test comparisons.

There was no significant main effect of test time (model fit improvement over the model containing random effects: χ^2^(1) = .793, *p* = .373, difference estimate = .022, *SE* = .025, *z* = .891, power = 0, 95% CI [0, .02]). As predicted, there was no significant effect of condition on overall performance (model fit improvement over the model containing random effects: χ^2^(1) = .138, *p* = .710, power = 0, 95% CI [0, .02], indicating participants in the marker and no marker groups performed at a statistically similar level overall (difference estimate = .051, *SE* = .138, *z* = .372), and there was no significant overall effect of consistency (model fit improvement over the model containing random effects: χ^2^(1) = .256, *p* = .613), indicating participants receiving consistent and inconsistent labeling performed at a statistically similar level overall (difference estimate = .070, *SE* = .137, *z* = .507). The critical interaction between condition and consistency was not significant, indicating that the transfer effect of distributional categorization dissipated over the course of the task, when representations between words and actions/objects were strengthened by the cross-situational statistics (model fit improvement when the interaction term was added to a model containing random effects plus main effects of condition and consistency: χ^2^(1) = 1.616, *p* = .204, difference estimate = −.346, *SE* = .270, *z* = −1.281, power = 0.32, 95% CI [.26, .39]. See Table 5 for a summary of this model.[Table-anchor tbl5]

### Transfer Task: Vocabulary Test

We examined the data from the vocabulary task to establish whether high frequency words influenced word learning during the cross-situational learning task, with subsequent analysis testing separately learning of nouns and verbs to ensure that both categories of word were acquired.

One-sample *t* tests were performed on the mean proportion of correct responses to compare overall vocabulary scores to chance (.5). Performance was significantly above chance for both groups (no markers: *M* = .653, *SE* = .033, *t*(23) = 4.674, *p* < .001; markers: *M* = .600, *SE* = .037, *t*(23) = 2.724, *p* = .012), further indicating that participants in both conditions were able to learn the mappings on the cross situational learning task.

Subsequent analysis examined the effect of consistency on vocabulary learning, to see whether the distributional categories influenced learners’ mapping of targets to their action/object referents: It is possible that alignment or conflict between distributional and grammatical categories may have helped or impeded word learning, respectively, as seen in the cross-situational learning task. Because consistency was only functionally relevant for the markers group, we performed separate analysis for the markers and no markers conditions and focus on the markers group here, and include the no markers analysis as an additional check.

Generalized linear mixed effects analysis was performed on the data for the markers condition, modeling response accuracy on the vocabulary test considering variation across participants and materials. The model was initially fitted with random effects of subjects, trial, particular items, and language version. We then sequentially added consistency (consistent/inconsistent) and word type (noun or verb) as fixed effects, followed by the interaction term, and considered their respective effects on model fit with likelihood ratio test comparisons. Due to a technical error, key-press responses were not recorded for eight out of the 384 observations for the markers group, and 12 out of the 384 observations for the no markers group (missing responses were approximately evenly distributed across consistent/inconsistent groups and noun/verb trials). These trials were excluded from analysis (see Tables 6 and 7 for a full breakdown of observations for each group).[Table-anchor tbl6][Table-anchor tbl7]

**Figure 4 fig4:**
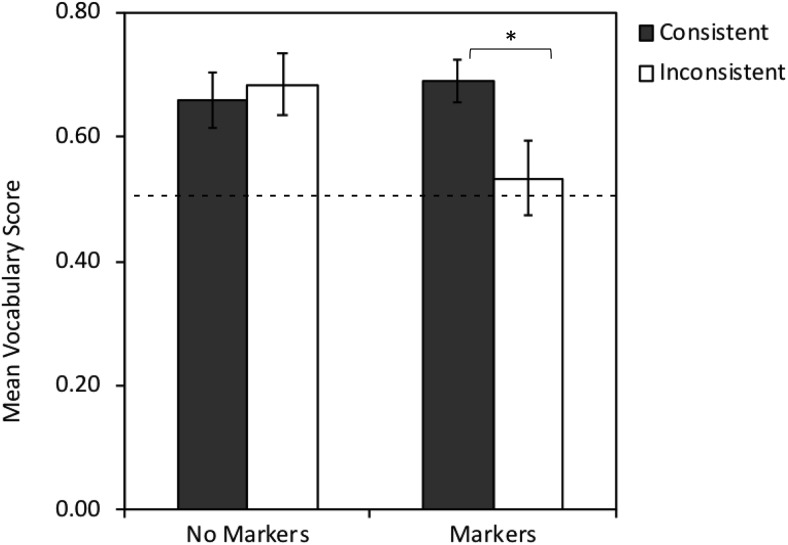
Mean scores (proportion correct) on the vocabulary task for participants receiving action/object labels that were either consistent or inconsistent with the trained grammatical distinctions, given for both conditions. Error bars indicate standard error. * Asterisks indicate statistical significance.

**Figure 5 fig5:**
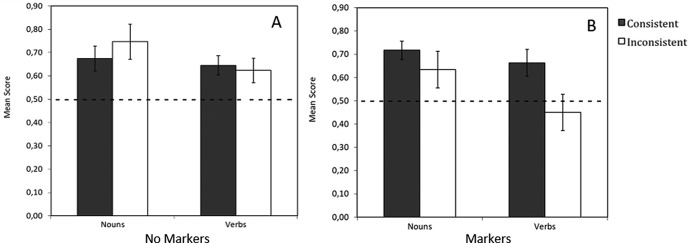
Mean vocabulary scores for the no markers (A) and markers (B) training conditions, given by consistency and word-type. Error bars indicate standard error. Because categories were denoted by marker words during training, consistency was only relevant for markers.

For the markers group, there was a significant effect of word type (model fit improvement over the model containing random effects: χ^2^(1) = 4.232, *p* = .040, difference estimate = −.543, *SE* = .241, *z* = −2.247, *p* = .025, power = .57, 95% CI [.49, .63]), with better learning for nouns (*M* = .670, *SE* = .044) than verbs (*M* = .555, *SE* = .052). There was a significant effect of consistency (model fit improvement when consistency was added to the model containing random effects plus word type: χ^2^(1) = 4.141, *p* = .042, difference estimate = −.539, *SE* = .242, *z* = −2.229, *p* = .026, power = .63, 95% CI [.56, .70]), with participants performing better when label use was consistent with the distributional categories (*M* = .689, *SE* = .034) compared with inconsistent (*M* = .534, *SE* = .060, see Figure 4). The interaction between word type and consistency was not significant (model fit improvement when the interaction term was added to a model containing random effects and main effects of word type and consistency: χ^2^(1) = 1.568, *p* = .211, difference estimate = −.619, *SE* = .516, *z* = −1.200, *p* = .230, power = 0, 95% CI [0, .02], suggesting the distributional cues affected learning of nouns and verbs equally. See Table 6 for a summary of the final model, and see Figure 5 for a visualization of the results for this task.[Fig-anchor fig4][Fig-anchor fig5]

The same analysis conducted for the no markers group found no significant effect of word type, though this was approaching significance (model fit improvement over the model containing random effects: χ^2^(1) = 2.755, *p* = .097, difference estimate = −.387, *SE* = .233, *z* = −1.658, *p* = .097, power = .41, 95% CI [.34, .48], with better learning for nouns (*M* = .710, *SE* = .044) than verbs (*M* = .634, *SE* = .052). As expected, there was no significant effect of consistency (model fit improvement over the model containing random effects: χ^2^(1) = .185, *p* = .667, difference estimate = .135, *SE* = .314, *z* = .431, *p* = .667, power = .12, 95% CI [.08, .17], with no difference in performance between participants receiving consistent (*M* = .660, *SE* = .044) versus inconsistent (*M* = .685, *SE* = .049) mappings (this was as anticipated, because these participants received no cues to category membership). There was no significant interaction between word type and consistency, χ^2^(1) = 1.232, *p* = .267; difference estimate = −.524, *SE* = .468, *z* = −1.111, *p* = .267; and power = 0, 95% CI [0, .02]. See Table 7 for a summary of the final model, and see Figure 5.

Building on the results seen at Test 1 of the transfer task, these data provide further evidence that learners’ language use was shaped by the interaction between the distributional categories denoted by the high frequency marker-words with the congruent or incongruent use of targets from these categories to label nouns and verbs; suggesting that grammatical categorization was influenced by the presence of high frequency marker words in the training speech.

## Discussion

We investigated the possibility that high frequency words in speech can assist speech segmentation while simultaneously informing distributional category formation (Monaghan & Christiansen, 2010; Monaghan et al., 2007). We also tested whether participants could map these distributional categories onto grammatical categories of words (nouns and verbs). The results support the suggestion that high-frequency marker words, previously shown to help speech segmentation (Bortfeld et al., 2005; Cunillera et al., 2010; Cunillera, Laine, Càmara, & Rodríguez-Fornells, 2016; Lany, 2014), also guide the formation of grammatical categories in early language learning, right at the point where speech segmentation is just being learnt.

Previous studies of speech segmentation have demonstrated that learners are able to use transitional probabilities to support identification of word candidates (Hay et al., 2011; Saffran et al., 1996), and can draw on previously known high-frequency words to assist in acquisition of words that are adjacent to them (Bortfeld et al., 2005; Cunillera et al., 2010). In the current study, we examined learning in the combined presence of both of these information sources, to determine whether statistical speech segmentation is still possible under conditions of increased complexity of the language structure.

Participants were able to identify target words from the speech stream—regardless of whether that stream comprised target words only, or target words plus marker words. This is especially noteworthy given the increased complexity of speech in the markers condition (i.e., speech with multiple types of words, and words of different lengths). Participants’ ability to recognize targets during testing in the absence of the marker words is consistent with prior demonstrations that high-frequency marker words can be used as anchor points for segmentation to occur around (Altvater-Mackensen & Mani, 2013; Bortfeld et al., 2005; Cunillera et al., 2010, 2016; Mersad & Nazzi, 2012; Monaghan & Christiansen, 2010). In this case, it is possible that the high frequency markers led to comparatively similar performance to the control group—despite the increased complexity of the signal. However, there was no significant benefit of the high frequency words on statistical segmentation. Of note, though, is that while the study was designed with sufficient power to detect the effect of transfer from segmentation to the word learning study, post hoc power analyses suggest that our study was underpowered with regard to revealing an effect of marker words on segmentation performance. A higher-powered replication would therefore be advantageous for the field.

The inclusion of monosyllabic high-frequency marker words and bisyllabic targets meant that, for the markers condition, the language comprised sequences of words of varying length. These data document a rare demonstration of adults’ ability to use statistical information only to identify words from a continuous artificial speech stream containing words of varying length; detecting segmentation under such conditions has previously proven challenging (e.g., Kurumada et al., 2013), and even claimed to be impossible without additional scaffolding (e.g., Johnson & Tyler, 2010). Nevertheless, participants in both conditions were able to identify target words from this language, and to a similar degree. Critically, our data also demonstrate that segmentation of markers and targets was possible despite the language containing novel markers (high frequency words), that were not previously known to participants (e.g., Cunillera et al., 2010).

While participants were shown to identify words in the speech stream (i.e., recognize target words as likely lexical candidates over nonword competitors), our data do not provide evidence that the ability to do so was enhanced by the presence of the marker words. A possible reason for this is that the study presented here poses a slightly different challenge to the learner than that documented in previous research: Our training phase is wholly implicit, and comprises a continuous stream from which learners must discern *both* markers and targets. Previous studies of the anchor word effect have often used high frequency words that participants were already familiar with prior to the study (e.g., Bortfeld et al., 2005), or that they were trained on explicitly in the initial stages of the experiment (e.g., Cunillera et al., 2016). In such studies, participants would not be tasked with discovering the high frequency words in the speech stream along with targets, unlike in the present study. It is possible that greater gains to the segmentation task would emerge with prior exposure to the high frequency marker words, thus the benefit of high frequency words for speech segmentation may only be evident after words reach a certain threshold of familiarity (thereby facilitating the interplay between top-down and bottom-up processing suggested by Conway et al., 2010). In future studies, testing knowledge of the high frequency words separately and correlating this with performance on the segmentation task would provide critical insight into this possibility.

We have described the measure of preference for words versus nonwords as a segmentation test; however, we would like to note that performance on this task does not necessarily require words to be isolated as word candidates in order to distinguish them from nonwords (spanning two target words that did not occur together during training). Performance could be based on familiarity of the sequences. Equally, learning from the languages with and without marker words could have been rather different, with marker words interpreted by listeners as either a part of the word (such as an affix), or as a function word marking their role. Models of speech segmentation make different predictions about how these marker words would be interpreted—either as isolated words (e.g., Monaghan & Christiansen, 2010), or, as is likely in the PARSER model, as an integral part of the word (e.g., Perruchet & Vinter, 1998). Future research with additional tests of preference for sequences both including and omitting marker words alongside target words would enable us to distinguish these alternatives. Though these are important considerations, the results nevertheless provide strong evidence that participants are able to consider the target word (either as an isolated word or as the root of a word appearing at test without its prefix) as part of the language over a sequence that comprised two portions of different words.

The two additional tests of category learning enable us to further interpret how the marker words affected processing of the speech. The first involved analyzing whether participants had explicit knowledge of the distributional category structure of the language, and was similar to research involving identification of distributional categories from statistical distributions of co-occurrence of words in speech (e.g., Frigo & MacDonald, 1998; Mintz, 2002; Reeder et al., 2013). Using a related task, Frost et al. (2016) demonstrated that when two categories of words were preceded by one marker word each, the distributional categories were detected by participants, with findings indicating that the high-frequency marker words available for speech segmentation (e.g., Cunillera et al., 2016) were also useful for determining the potential category structure of the language. However, we did not replicate this categorization effect here. Sensitivity to distributional categories is often difficult to detect in artificial language learning studies unless multiple distributional cues (e.g., Mintz, 2002; St. Clair et al., 2010) or multiple cues from different modalities (e.g., Monaghan et al., 2005) are present in speech (and see also Ramscar, 2013 and St. Clair, Monaghan, & Ramscar, 2009 regarding the optimality of preceding vs. succeeding category markers for words). Providing just a preceding distributional cue is thus a weak (albeit usable) cue to distributional categories in the language. Since the data from the proceeding tasks indicate that participants did form some level of distributional categories, another possibility is that the task employed here did not adequately tap into participants’ implicit knowledge of distributional categories, due to its requirement for reflection-based responses (Christiansen, 2018; Isbilen, Frost, Monaghan, & Christiansen, 2018). In addition, power analyses indicated that a larger number of participants and/or test stimuli may be required to detect an effect of explicit distributional category knowledge.

The complexity of the artificial language we used served a dual function for learning—potentially supporting speech segmentation, and also determining the possible grammatical roles of words in the language at the point at which they are first identified from speech. Reeder et al. (2013) refer to distributional contexts such as those defined by the marker words in our language as “syntactic form-classes,” and they provide a potential precursor to grammatical categories in their relation to semantic features of their referents. The second test of category learning, the transfer task, enabled us to test whether participants were able to map their knowledge about distributional categories onto grammatical distinctions between nouns and verbs. This task also enabled us to determine whether this distributional information was available to support syntactic categorization at an early point in language learning—when speech is being segmented (Frost & Monaghan, 2016).

The high frequency marker words resulted in distributional categorization—evident in the early stages of the transfer task, and suggested also in the subsequent vocabulary task. Participants’ ability to learn the referents for words in different grammatical categories in a cross-situational learning task was affected by their prior exposure to distributional categories in the continuous language. In particular, performance was inhibited when mappings violated the distributional categories that participants had formed in training. The data therefore provide evidence that the formation of grammatical categories could be shaped by high frequency marker words (e.g., articles, pronouns) which often precede content word categories in speech (cf. Monaghan & Christiansen, 2010), and further demonstrate that this information is available for language learning at the very point at which words are beginning to be extracted from continuous speech. In future work on this topic, matching participants on their segmentation performance before assessing categorization, and collecting additional measures of individual differences, would help further unpack the relationship between these tasks in language acquisition.

Interestingly, the transfer effects arising from participants’ distributional category knowledge reduced over the course of the transfer task, as exposure to the cross-situational word-referent mappings increased. This indicates that the distributional categories only provided an advantage (or disadvantage, where these were inconsistent) early in learning mappings between words and referents belonging to different grammatical categories. Language learners are known to adapt to artificial language structure extremely quickly (e.g., Gerken, 2010) and participants receiving inconsistent mappings quickly caught up with participants who had experienced consistent mappings between distributional and grammatical categories across the tasks, with individual mappings strengthened with further exposure. Although the effects were seen to dissipate, results nevertheless indicate that participants’ prior learning of distributional categories did influence learning in the early stages of this task, as predicted based on Frost et al.’s (2016) pilot study. Though power was adequate for a high likelihood of finding the effect early in training, a higher-powered future replication would help shed further light on the precise pattern of learning on the cross-situational word learning transfer task.

Though the effects of the marker words in forming distributional categories diminished as the transfer task training proceeded, at the end of training the effect of this prior learning was still evident. In the vocabulary task for the markers condition, the effect of consistency was significant, with better learning when words were congruent with the category defined by the marker words than when they were incongruent. Thus, though quantitatively effects of learning were not observed as training continued, the qualitative effects of the marker words persistently exerted an effect on participants’ learning.

Previous computational models of segmentation and of grammatical category learning have (separately) shown that the same high-frequency words prove useful to each of these tasks (Monaghan & Christiansen, 2010). In this study, we have shown that the same high-frequency words can be accommodated to both speech segmentation and grammatical categorization. Our results therefore add further support to the view that these tasks are not temporally distinct, but rather may operate simultaneously from the very earliest stages of language learning (Frost & Monaghan, 2016).

## Supplementary Material

10.1037/xlm0000683.supp

## Figures and Tables

**Table 1 tbl1:** Example Speech Streams for Each Condition

Condition	Target words	Marker words	Speech stream excerpt
No markers	*samu, noli, fezu, nide, tero, buza, kato, mave*		*. . . tero-noli-nide-buza-fezu-mave-samu-kato-noli . . .*
Markers	”	*fo*, ***re***	. . . ***re***-*tero*-*fo*-*noli*-*fo*-*nide*-***re***-*buza*-*fo*-*fezu*-***re***-*mave*-*fo*-*samu*-***re***-*kato*-*fo*-*noli* . . .
*Note*. Items in grey belong to Category A, whereas items in black belong to Category B. Marker words are given in bold. Dashes indicate word boundaries, but these were not physically denoted in the continuous speech; items followed each other directly with no pauses between words.			

**Table 2 tbl2:** Summary of the Linear Mixed-Effects Model of (Log Odds) Segmentation Scores

			Wald confidence intervals			
Fixed effects	Estimated coefficient	*SE*	2.50%	97.50%	*z*	Pr (>|z|)
(Intercept)	.9887	.2045	.5879	1.3895	4.835	*p* < .001
Random effects	Variance	Std. Dev.				
Subject (Intercept)	.5721	.7563				
Test pair (Intercept)	.1757	.4192				
Language version (Intercept)	.0000	.0000				
	AIC	BIC	logLik	Deviance		
	903.4	921.9	−447.7	895.4		
*Note.* 768 observations, 48 participants, 16 trials. R Syntax for the final model is: aw_seg1 < − glmer (accuracy ∼ (1|subject) + (1|test_pair) + (1|lang_version), data = anchor2_seg, family = binomial).						

**Table 3 tbl3:** Summary of the Linear Mixed-Effects Model of Similarity Ratings on the Categorization Task

			Wald confidence intervals		
Fixed effects	Estimated coefficient	*SE*	2.50%	97.50%	*t* value
(Intercept)	3.5191	.1170	3.2898	3.7484	30.077
Condition	.3559	.1631	.0362	.6756	2.182
Random effects	Variance	Std. Dev.			
Subject (Intercept)	.2416	.4916			
Trial (Intercept)	.0092	.0961			
Language version (Intercept)	.00003	.0053			
	AIC	BIC	LogLik	Deviance	
	4069.9	4100.2	−2029.0	4057.9	
*Note.* 1,152 observations, 48 participants, 24 trials. R syntax for the final model is: aw_cat1R2 < − lmer (rating ∼ condition + (1|subject) + (1|trial) + (1|version), data = a2_cat1, REML = TRUE).					

**Table 4 tbl4:** Summary of the Generalized Linear Mixed-Effects Model of (Log Odds) Accuracy Scores on Block 1 of the Transfer Task

			Wald confidence intervals			
Fixed effects	Estimated coefficient	*SE*	2.50%	97.50%	*z*	Pr (>|z|)
(Intercept)	−.3687	.2976	−.9520	.2146	−1.239	.2154
Condition	.9764	.3751	.2412	1.7116	2.603	.009
Consistency	.6462	.3711	−.0812	1.374	1.741	.0817
Condition × Consistency	−1.4358	.5282	−2.4731	−.4024	−2.722	.0065
Random effects	Variance	Std. Dev.				
Subject (Intercept)	.2612	.5111				
Trial (Intercept)	9.483 × 10^−10^	3.080 × 10^−5^				
Target 1 (Intercept)	.1584	.3980				
Target 2 (Intercept)	5.868 × 10^−9^	7.660 × 10^−5^				
Lang_v (Intercept)	3.838 × 10^−10^	1.957 × 10^−5^				
Word order	9.272 × 10^−9^	9.629 × 10^−5^				
	AIC	BIC	LogLik	Deviance		
	532.2	571.7	−256.1	512.2		
*Note.* 384 observations, 48 participants, eight trials. R syntax for the final model is: aw_xsit5 < − glmer (accuracy ∼ condition × consistency + (1|subject) + (1|lang_v) + (1|target1) + (1|target2) + (1|wordorder) + (1|trial), data = anchor2_xsit_items_BLOCK1, family = binomial).						

**Table 5 tbl5:** Summary of the Generalized Linear Mixed-Effects Model of (Log Odds) Accuracy Scores on the Transfer Task

			Wald confidence intervals			
Fixed effects	Estimated coefficient	*SE*	2.50%	97.50%	*z*	Pr (>|z|)
(Intercept)	.1428	.1456	−.1425	.4281	.981	.327
Condition	.2232	.1906	−.1504	.5969	1.171	.242
Consistency	.2417	.1905	−.1317	.6151	1.269	.205
Condition × Consistency	−.3457	.2699	−.8747	.1832	−1.281	.200
Random effects	Variance	Std. Dev.				
Subject (Intercept)	.1303	.3610				
Target 1 (Intercept)	.0000	.0000				
Target 2 (Intercept)	.0120	.1096				
Lang_v (Intercept)	4.038 × 10^−14^	2.010 × 10^−7^				
Word order	.0034	.0578				
	AIC	BIC	LogLik	Deviance		
	3134.8	3186.5	−1558.4	3116.8		
*Note.* 2,304 observations, 48 participants, 48 trials. R syntax for the final model is: aw_xsitA8 < − glmer (accuracy ∼ condition × consistency + (1|Subject) + (1|lang_v) + (1|Target1) + (1|Target2) + (1|WordOrder), data = anchor2_xsit_items, family = binomial, control = glmerControl (optimizer=“bobyqa”, optCtrl = List(maxfun = 100000))).						

**Table 6 tbl6:** Summary of the Generalized Linear Mixed-Effects Model of (Log Odds) Accuracy Scores on the Vocabulary Test for the Markers Group

			Wald confidence intervals			
Fixed effects	Estimated coefficient	*SE*	2.50%	97.50%	*z*	Pr (>|z|)
(Intercept)	1.1385	.2725	.6044	1.6725	.981	2.93 × 10^−5^
Word type	−.5389	.2417	−1.0127	−.0651	−2.229	.0258
Consistency	−.6983	.3274	−1.340	−.0567	−2.133	.0329
Random effects	Variance	Std. Dev.				
Subject (Intercept)	.3365	.5801				
Target (Intercept)	.0142	.1193				
Trial (Intercept)	.0000	.0000				
Lang_v (Intercept)	.0000	.0000				
	AIC	BIC	LogLik	Deviance		
	493.0	520.5	−239.5	479.0		
*Note.* 376 observations (nouns: 185, verbs: 191; consistent: 187; inconsistent: 189), 48 participants, 48 trials. R syntax for the final model is: aw_vocabMF4 <− glmer (accuracy ∼ consistency + word_type + (1|Subject) + (1|target) + (1|lang_v) + (1|trial), data = a2_vocab_MARKERS_F, family = binomial, control = glmerControl (optimizer=“bobyqa”, optCtrl = list (maxfun = 100000))).						

**Table 7 tbl7:** Summary of the Generalized Linear Mixed-Effects Model of (Log Odds) Accuracy Scores on the Vocabulary Test for the No Markers Group

			Wald confidence intervals			
Fixed effects	Estimated coefficient	*SE*	2.50%	97.50%	*z*	Pr (>|z|)
(Intercept)	.7911	.1852	.4280	1.1542	4.27	1.95 × 10^−5^
Random effects	Variance	Std. Dev.				
Subject (Intercept)	.2738	.5232				
Target (Intercept)	.0000	.0000				
Trial (Intercept)	.1363	.3692				
Lang_v (Intercept)	.0000	.0000				
	AIC	BIC	LogLik	Deviance		
	493.0	520.5	−239.5	479.0		
*Note.* 372 observations (nouns: 186, verbs: 186; consistent: 188; inconsistent: 184), 48 participants, 48 trials. R syntax for the final model is: aw_vocabNO_F <− glmer (accuracy ∼ (1|Subject) + (1|target) + (1|lang_v) + (1|trial), data = a2_vocab_NO_F, family = binomial, control = glmerControl (optimizer = “Bobyqa”, optCtrl = list(maxfun = 100000))).						

## Footnotes

1 Note that even in the case of putative innate categories (whether they be syntactic or semantic), there is still the problem of mapping individual sound patterns from a language onto those categories (e.g., the word *penguin* onto the category of nouns). The kind of distributional learning described here might therefore also be needed in theories positing pre-existing innate categories.
