# Supplementary material for: Mark My Words: High Frequency Marker Words Impact Early Stages of Language Learning
Source: J Exp Psychol Learn Mem Cogn. 2019 Jan 17;45(10):1883–98. doi: 10.1037/xlm0000683 (PMC6746567; doi:10.1037/xlm0000683)
Supplement: Supplementary file 1 [file XLM-2018-0106_High-frequency-words.docx]

**Supplementary Materials**

**Segmentation test**

To permit direct comparison with prior work on this topic, an ANOVA with condition and language version as factors was performed on the segmentation data. There was no significant effect of condition, *F* (1, 36) = 2.670, *p* = .111, η_p_^2^ = .069, post hoc power = .46, with participants in both conditions performing better than chance, but not significantly different than one another (see conference proceedings by Frost et al., 2016, for a similar observation).


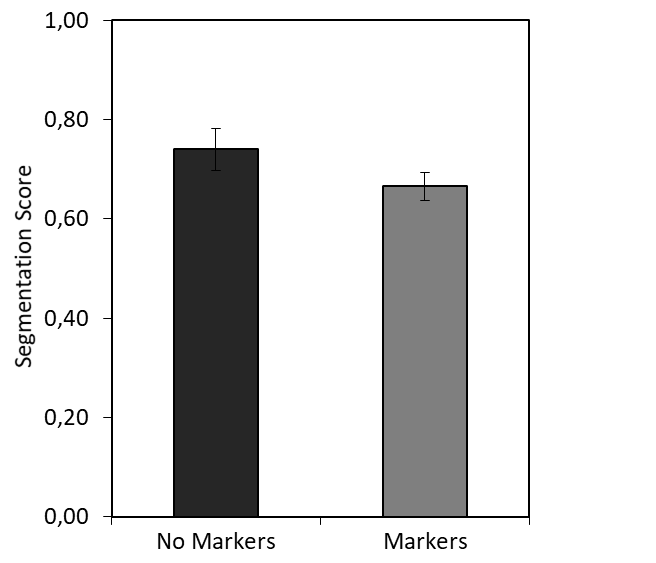


*

*

Supplementary Figure i. Mean accuracy scores on the segmentation task for each condition, with SE. Asterisks indicate above chance performance.


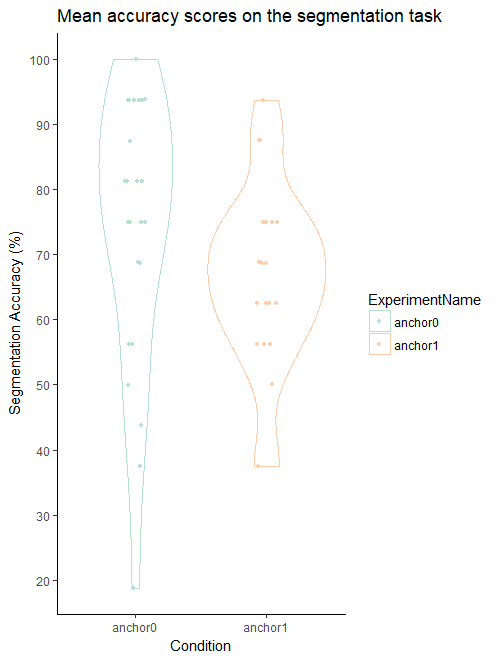


No Markers

Markers

No Markers

Markers

Condition

Condition

Supplementary Figure ii. Violin plot illustrating the distribution of accuracy scores on the segmentation task for each condition.

**Categorisation Test**

To permit comparison with the pilot work on this topic (see Frost, Monaghan & Christiansen, 2016), a repeated-measures ANOVA was performed, with test-pair type (same or different category) as a within-subjects factor, and marker condition (No Markers, Markers) and language version as between-subjects factors. There was a significant effect of condition, *F* (1, 36) = 4.712, *p* = .037, η_p_^2^ = .116, power = .94, with the No Markers group giving significantly higher similarity ratings overall compared with the Markers group, as outlined in the main text. However, there was no significant effect of test-pair type, *F* < 1, and there was no significant interaction between test-pair type and marker condition, *F* < 1.

Test pair type

Supplementary Figure iii. Mean similarity ratings (1-6) for test pairs containing items taken from the same versus different grammatical categories, given for each condition.

**Transfer of Category Knowledge Task**

In order to determine whether distributional categories (formed based on the co-occurrence of markers and targets) influenced grammatical category formation, a univariate ANOVA was performed on the data for the first test. Since only the Markers group received distributional category cues, and only the inconsistent group encountered conflict between distributional categories and the categories on this task, transfer effects here would be evidenced through an *interaction* between these two variables. There was no significant effect of marker condition, and there was no significant effect of consistency (both *F* < 1), but, critically, the interaction between these two variables was significant, *F* (1, 44) = 6.845, *p* = .012, η_p_^2^ = .135, power = .76, indicating that prior knowledge about distributional categories influenced early performance on this task.

Subsequent analysis examined the way that distributional categorisation affected performance across the task as a whole. A repeated-measures ANOVA was performed with marker condition (No Markers, Markers), consistency (consistent, inconsistent), and language version as a between subjects factors, and test time (test1, test2, test3, test4, test5, test6) as a within subjects factor. There was no significant effect of test time, *F* (5, 120) = 1.083, *p* = 0.373, η_p_^2^ = .043, power = .79. There was no significant effect of marker condition, *F* < 1 and there was no significant interaction between test time and marker condition, *F* < 1. There was no significant effect of consistency, *F* < 1, and the critical interaction between consistency and condition was not significant, *F* (1, 24) = 1.315, *p* = .263, η_p_^2^ = .052, indicating that the transfer effects dissipated over the course of the task, with cross-situational associations strengthening to outweigh the distributional categories (see the conference proceedings by Frost et al., 2016, for a similar pattern of results but with a word-picture mapping task).

**
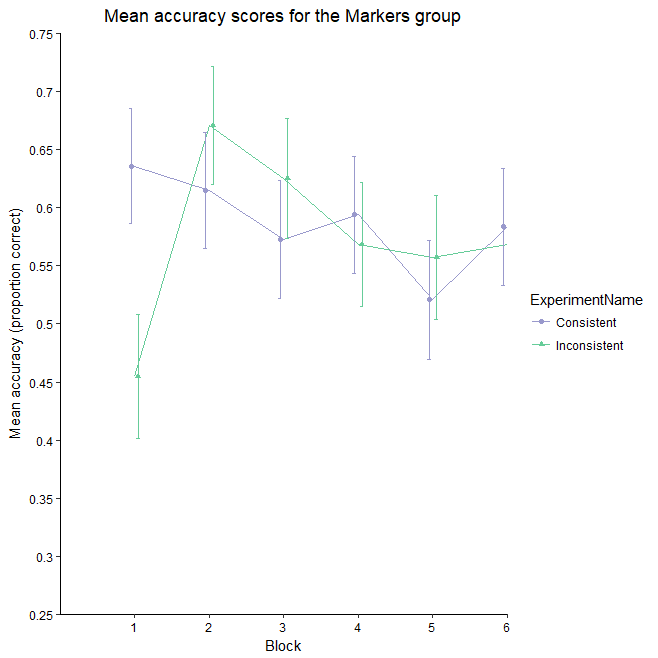
**


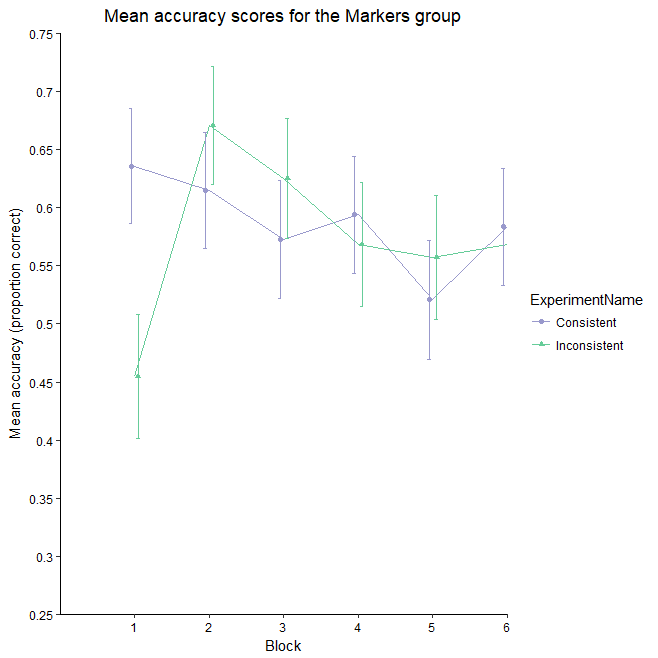


Consistency

Supplementary Figure iv. Mean accuracy scores over the course of the transfer task for the Markers group, given for each labelling condition.


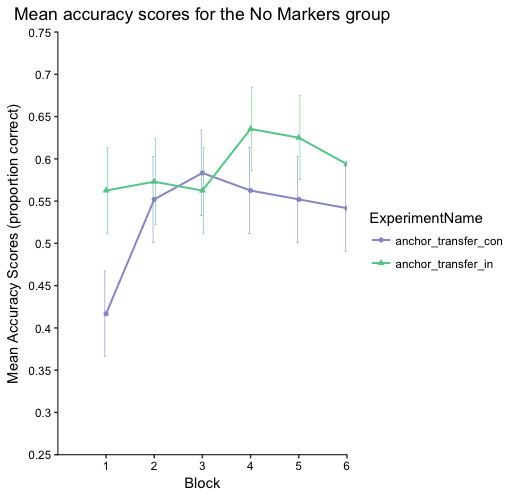


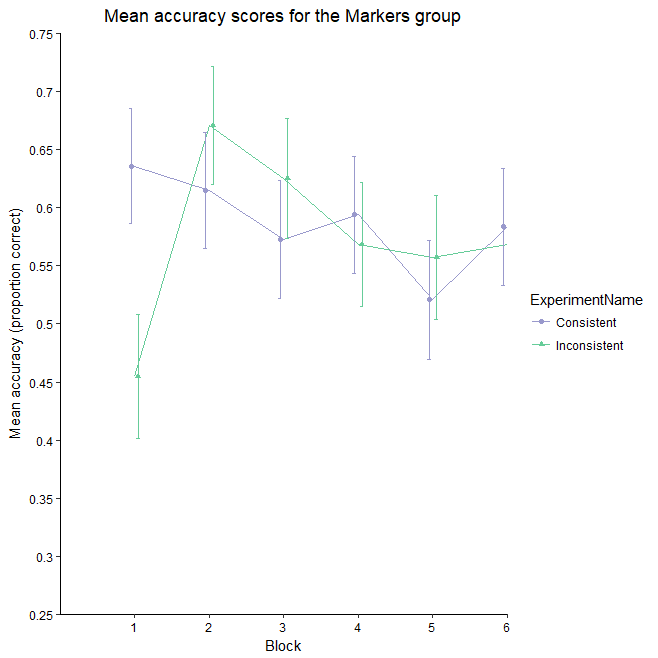


Consistency

Supplementary Figure v. Mean accuracy scores over the course of the transfer task for the No Markers group, given for each labelling condition.


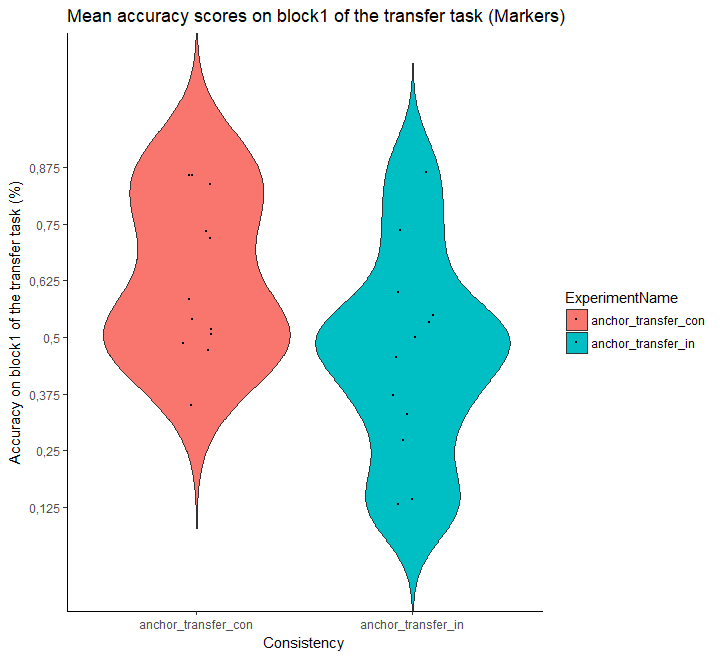


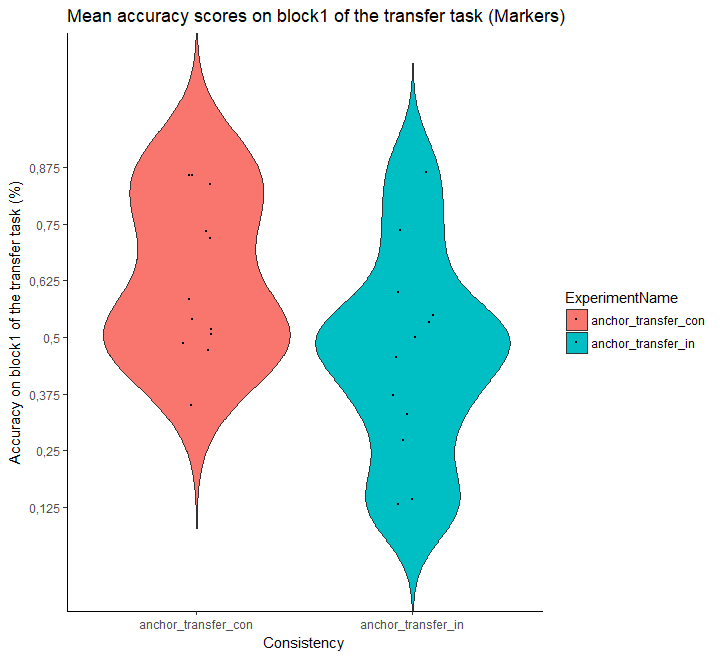


Consistent

Consistency

Inconsistent

Consistent

Inconsistent

Supplementary Figure vi. Violin plot illustrating the distribution of scores at block1 for the Markers group, for each labelling condition.


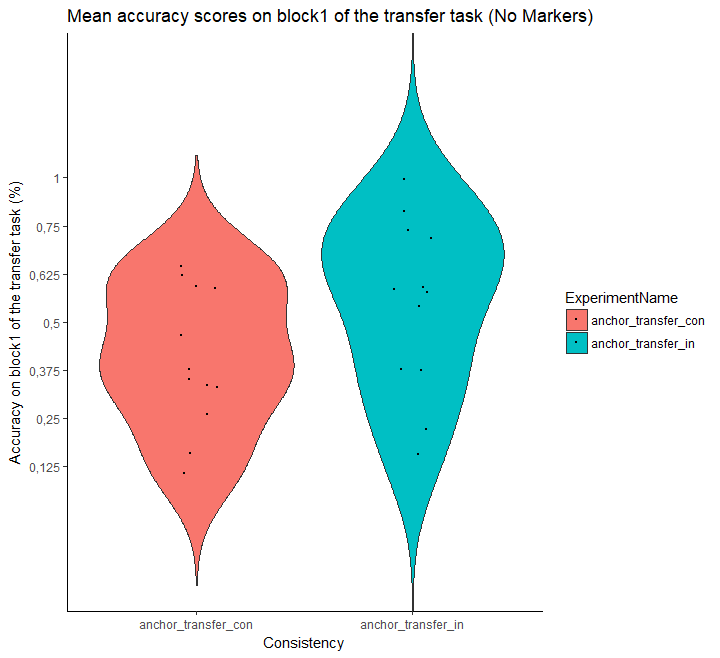


Consistent

Inconsistent


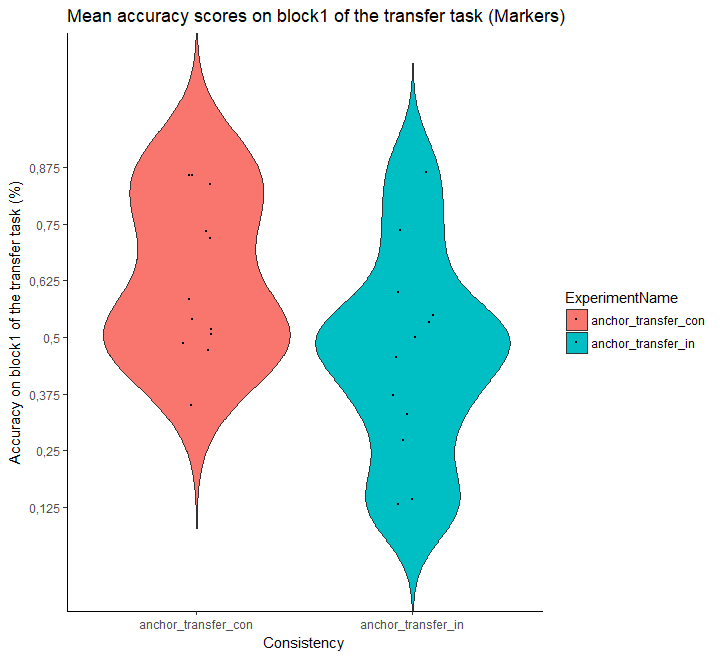


Consistent

Consistency

Inconsistent

Supplementary Figure vii. Violin plot illustrating the distribution of scores at block1 for the No Markers group, for each labelling condition.

**Vocabulary Test**

A 2x2 repeated measures ANOVA performed on the data for the Markers condition, with consistency (between subjects) and word type (within subjects) as factors. There was no significant effect of word type, *F* (1, 22) = 2.338, *p* = .141, η_p_^2^ = .096, power = .58, though trends in the means indicated learning was better for nouns than verbs. There was a significant effect of consistency, *F* (1, 22) = 4.515, *p* = .045, η_p_^2^ = .170, power = .85, with participants performing better when labels were used consistently with their distributional category compared to inconsistently. The interaction between word type and consistency was not significant, *F* (1, 22) = 1.034, *p* = .320, η_p_^2^ = .045, power = .30, suggesting the distributional cues affected learning of nouns and verbs equally.

The same 2x2 ANOVA conducted for the No Markers group found no significant effect of word type, *F* (1, 22) = 2.543, *p* = .125, η_p_^2^ = .104, power = .62, though again trends in the means indicated better learning for nouns than verbs. As expected, there was no significant effect of consistency (*F* < 1), with no difference in performance between participants receiving consistent versus inconsistent mappings (this was as anticipated, since these participants received no cues to category membership). The interaction between word type and consistency was not significant (*F* < 1).
